# Prognostic relevance of exercise testing in hypertrophic cardiomyopathy. A systematic review

**DOI:** 10.1016/j.ijcard.2021.06.051

**Published:** 2021-09-15

**Authors:** Tiago Rodrigues, Sofia Cavaco Raposo, Dulce Brito, Luis R. Lopes

**Affiliations:** a Cardiology Department, Centro Hospitalar Universitário Lisboa Norte, Av. Prof. Egas Moniz, 1649-028 Lisboa, Portugal; b CAML, CCUL, Lisbon School of Medicine, Universidade de Lisboa, Av. Prof. Egas Moniz, 1649-028 Lisboa, Portugal; c Unidade de Saúde Familiar Reynaldo dos Santos, Agrupamento de Centros de Saúde Estuário do Tejo, Administração Regional de Saúde Lisboa e Vale do Tejo, Portugal; d Lisbon School of Medicine, Universidade de Lisboa, Lisboa, Portugal; e Barts Heart Centre, St. Bartholomew's Hospital, London, UK; f Centre for Heart Muscle Disease, Institute of Cardiovascular Science, University College London, London, UK

**Keywords:** Systematic review, Hypertrophic cardiomyopathy, Exercise testing, Prognostic stratification

## Abstract

**Background:**

Cardiopulmonary exercise test (CPET) is indicated as part of the assessment in hypertrophic cardiomyopathy (HCM) patients and stress echocardiography is often used to assess symptoms. However, the role of exercise testing for prognostic stratification in HCM is still not established.

**Aims:**

To systematically review the evidence on the role of exercise testing for prognostic stratification in hypertrophic cardiomyopathy.

**Methods:**

A systematic review was conducted for eligible publications, between 2010 and 2020, that included evaluation of outcomes and prognosis. In these studies, patients underwent exercise echocardiography and/or cardiopulmonary exercise testing, performed according to predefined protocols. Diverse parameters were assessed in order to determine which were relevant for the prognosis. Analyzed outcomes included death from any cause, sudden cardiac death (SCD) and equivalents, cardiovascular death, heart failure requiring hospitalization or progression to New York Heart Association classes III or IV, cardiac transplantation, non-sustained ventricular tachycardia, stroke, myocardial infarction and invasive septal reduction therapy.

**Results:**

Eighteen publications were included, corresponding to a total of 7525 patients. The mean follow-up period varied between 1 and 8 years. The main findings of these studies revealed that the major predictors of outcomes were abnormal heart rate recovery, abnormal blood pressure response exercise induced wall motion abnormalities, lower peak VO2, higher VE/VCO2, and pulmonary hypertension/exercise-induced pulmonary hypertension.

**Conclusion:**

Although most studies concluded that exercise test results are useful to determine prognosis in HCM, further investigation is needed regarding whether it adds independent value to the current risk stratification strategies.

## Highlights

• HCM patients are frequently limited or initially present with reduced exercise capacity
• The role of exercise testing for prognostic stratification in HCM is still not established in the guidelines
• Exercise echocardiography or cardiopulmonary exercise testing are able to evaluate functional status and risk stratify HCM

## 1 Introduction

Hypertrophic Cardiomyopathy (HCM) affects approximately 0.2% of the adult population worldwide. It is characterized by left ventricular hypertrophy (LVH) in the absence of another cardiac, systemic, or metabolic disease capable of producing the magnitude of hypertrophy seen; a causal sarcomere (or sarcomere-related) variant might be identified, or a genetic etiology may remain unresolved [[1], [2], [3]]. It is a cause of sudden cardiac death (SCD), heart failure (HF) and arrhythmias including atrial fibrillation [1].

Patients frequently present with reduced exercise capacity [[1], [2], [3]], which can be explained by diverse mechanisms, including left ventricular outflow tract obstruction (LVOTO), myocardial ischemia and left ventricular systolic and diastolic dysfunction [1]. Chest pain is also a common presenting symptom.

Cardiopulmonary exercise testing (CPET) is recommended as part of the initial evaluation of patients with a diagnosis of HCM, when they report change in symptoms or when considering therapy to reduce LVOTO [1,[2], [3]]. It allows the measurement of peak oxygen consumption (VO2), minute ventilation relative to CO2 production (VE/VCO2) slope, aerobic threshold (first ventilatory threshold - where the lactate production rate is higher than the metabolizing capacity of the muscle cell) and anaerobic threshold (second ventilatory threshold – defined when the muscular lactate production rate exceeds the systemic lactate elimination rate) [4,5]. It may clarify equivocal symptoms, evaluate functional capacity prior to corrective therapeutic procedures, help distinguish between cardiac and pulmonary etiologies of functional impairment [1,6]. In addition, it was suggested it may allow a possible incremental prognostic role in the low-intermediate SCD risk categories over the contemporary strategies [3].

Exercise echocardiography allows evaluation of systolic anterior motion of the mitral valve, mitral regurgitation, left ventricular outflow tract (LVOT) or mid-cavity pressure gradients, diastolic and systolic function while on cycloergometer or treadmill exercise, providing valuable information to explain and assess the degree of severity of some of the symptoms [1,6].

Overall, ECG exercise testing, CPET and exercise echocardiography have been reported to predict multiple aspects of the prognosis in HCM patients. However, current guidelines do not incorporate any type of exercise testing for risk stratification strategies in HCM [1].

The aim of this work was to systematically review the current evidence concerning the relevance of these three forms of exercise testing for prognostic assessment in hypertrophic cardiomyopathy.

## 2 Methods

### 2.1 Search strategy and study selection

A systematic search was made on December 2020, using the Medline/PubMed and Embase databases, years 2010–2020, as well as searching for the publications included in the bibliography of the selected articles. The keywords used were: “prognosis AND (exercise OR stress OR cardiopulmonary) AND hypertrophic cardiomyopathy”.

This systematic review of the literature was conducted using the methodology suggested by the Preferred Reporting Items for Systematic Reviews and Meta-Analysis (PRISMA) guidelines [7]: a comprehensive search of the literature to identify all potentially relevant studies; systematic selection of studies based on inclusion and exclusion criteria; and extraction of relevant data from eligible studies.

The following inclusion criteria were applied: prospective or retrospective studies; performed in humans; participants older than 18 years; published in English; study population fulfilling the diagnostic criteria for HCM; making use of electrocardiography, echocardiography or cardiopulmonary exercise tests; evaluation of outcomes and prognosis.

Primary search was performed by two authors, who independently reviewed each reference (title and abstract) identified by the literature search, applied the inclusion and exclusion criteria listed above and decided on whether to include or exclude the publication at that stage. Any disagreement was solved by the senior author.

### 2.2 Data extraction

#### 2.2.1 Patients and baseline assessment

Inclusion of patients required a diagnosis of HCM based on guidelines criteria [1]. Baseline patient characteristics that were analyzed were: age [[8], [9], [10], [11], [12], [13], [14], [15], [16], [17], [18], [19], [20], [21], [22], [23], [24], [25]]; the presence of cardiovascular symptoms (dyspnoea, pre-syncope, syncope, angina, palpitations) [8,10,11,[13], [14], [15], [16], [17], [18], [19], [20], [21], [22], [23], [24]] or disease (coronary disease, hypertension, arrhythmia, valve disease) [[8], [9], [10], [11], [12],[14], [15], [16],[20], [21], [22],^24^,^25^], as well as other systemic diseases (genetic, metabolic) that could cause myocardial hypertrophy [9,10,13,14,16,21]; family history of premature sudden death or HCM [[8], [9], [10], [11], [12],[14], [15], [16], [17],^21^,^24^]; medication at the time of the study [[8], [9], [10], [11], [12],[14], [15], [16],[20], [21], [22], [23], [24], [25]]; history of cardiac surgery and/or medical therapy to relieve LVOTO [[9], [10], [11],^15^,^21^,^25^]; an adequate acoustic window [14,21] for echocardiographic studies and the ability and will to undergo exercise testing [14,21].

Other relevant initial baseline assessments included New York Heart Association (NYHA) functional class of HF [8,9,[11], [12], [13],[15], [16], [17], [18], [19], [20], [21], [22], [23], [24]], 12‑lead ECG at rest [8,10,12,15,17,20] and 24 h-ECG [8].

#### 2.2.2 Resting and exercise echocardiography

In some studies [[8], [9], [10], [11], [12], [13], [14], [15], [16], [17], [18], [19], [20], [21], [22], [23], [24], [25]], patients underwent resting transthoracic echocardiography, including M-Mode, bi-dimensional (2D) and, in some cases, Doppler evaluation, followed by symptom-limited exercise echocardiography in a treadmill in four studies [12,14,17,20,21].

Regarding exercise echocardiography [12,14,17,[20], [21], [22]], medications were not generally withdrawn before the test. Heart rate (HR) and blood pressure (BP) were measured during the test and afterwards, and ECG was monitored.

#### 2.2.3 Cardiopulmonary exercise testing

In those studies in which patients were submitted to CPET [10,11,[13], [14], [15], [16],^20^,^22^,^24^], the tests were performed with regular HR and BP measurements and ECG monitoring, according to standard protocols (*e.g.* Bruce), with gas exchange measurement.

Heart rate recovery (HRR), defined as a drop in heart rate (HR) from peak to 1 min post-exercise [26] was measured in two of the studies [12,15], to ascertain the fraction of patients with abnormal HRR (<12 beat drop over 1 min in recovery). Abnormal BP response (defined as progressive hypotension or a failure to increase the systolic blood pressure > 20 mmHg during exercise [27]) was also recorded in eight studies [[10], [11], [12], [13],^15^,^17^,^18^,^20^,^22^,^23^].

#### 2.2.4 Follow-up and outcomes

Patients were followed up with a defined regularity, so as to determine the occurrence of events. Death certificates were also analyzed in three of the studies [12,14,22].

Primary outcomes of relevance were death from any cause [[8], [9], [10],^12^,^14^,^16^,^18^,^20^,^21^,^23^], sudden death (defined as unexpected sudden collapse occurring <1 h from the onset of symptoms in patients who had previously experienced a relatively stable course [18,[23], [24]].), death due to progressive HF or “other” [8,9,11,14,16,20,21,23], sudden death equivalents (successful resuscitation from cardiac arrest and appropriate implantable cardioverter defibrillator (ICD) discharge), heart failure (HF) requiring hospitalization [9,11,16,20,21] or progression from NYHA class I or II to class III or IV of HF and cardiac transplantation [14,15,20,23,24].

Secondary outcomes of relevance were sustained ventricular tachycardia [8,12,14,18,21]; stroke in the context of atrial fibrillation (AF) [12,14,16,21]); myocardial infarction [14] and clinical deterioration leading to need of therapy to relieve LVOTO (including septal reduction) [14].

## 3 Results

### 3.1 Study selection

**Fig. 1:**
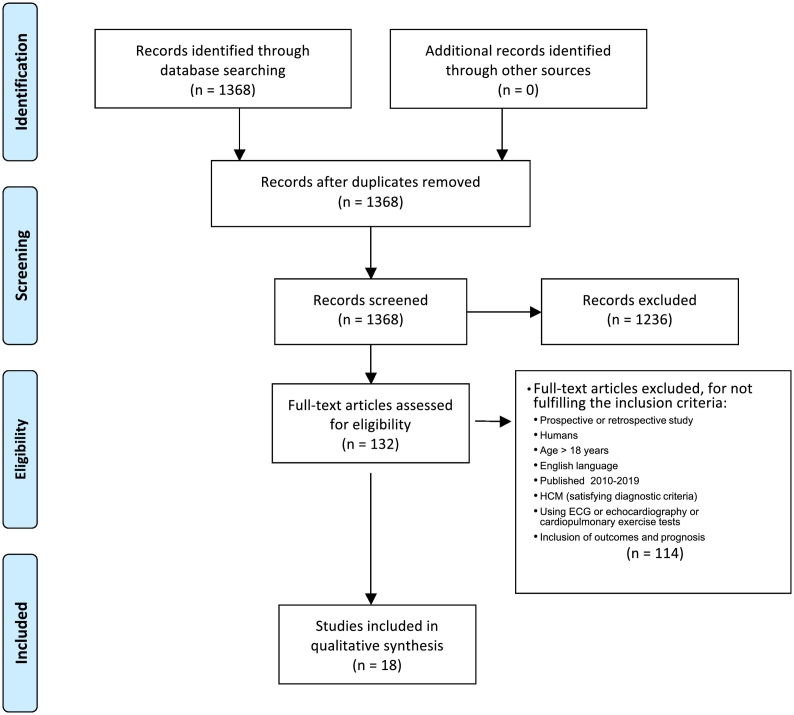
Study selection flow chart.

1368 unique publications were found and analyzed. 1236 of these publications were excluded for not fulfilling the age, date of publication and language criteria. The remaining 132 were analyzed by reading the abstract, and 114 of them excluded for not fulfilling the remaining criteria. Therefore, 18 studies were selected for this systematic review [[8], [9], [10], [11], [12], [13], [14], [15], [16], [17], [18], [19], [20], [21], [22], [23], [24], [25]] (Fig. 1), corresponding to a total of 7525 patients.

### 3.2 Design of the studies and baseline characteristics of the patients

Most studies (16 out of 18) were single-centre and observational. Only two studies included over 1000 patients [15,17]. The mean follow-up was between 1.6 ± 0.95 years and 8.7 ± 3 years. Mean age of the patient populations varied between 44 ± 14 and 59 ± 21 years. A percentage between 33 and 78.1 were men, with a positive familial history of HCM present in at least 20% up to 51% of the study population. Up to 22% of the patients were in class NYHA>II at baseline. Between 5% and 20.5% of patients had a diagnosis of AF and 20% to 42% had hypertension. The assessment and reporting of “classical” risk factors for sudden death, *i.e.* defined according to the 2003 ESC/American College of Cardiology (ACC) [1] and 2011 ACC/AHA (American Heart Association) guidelines [2] such as syncope, family history of SCD, non-sustained ventricular tachycardia (NSVT), abnormal BP response to exercise, and LV wall thickness ≥ 30 mm varied, but were generally present in less than 20% of the study population in almost all studies. The ESC clinical risk prediction model for sudden cardiac death in HCM patients considers eight different predictors (age, maximal left ventricular wall thickness, left atrial diameter, left ventricular outflow tract gradient, family history of SCD, non-sustained ventricular tachycardia, and unexplained syncope) to provide individualized 5-year risk for sudden cardiac death. In the analyzed studies these characteristics were underreported, limiting calculation of this score for the studies published after the score - and more recent European guidelines - were published [1,28]. The percentage of patients under β-blockade was between 33.1% and 90%. A minority of patients had, at baseline, a permanent pacemaker (5 to 21% of the population in the different studies) or an ICD (0 to 39% of the population). Baseline characteristics of the patients included in the studies are summarized in Table 1, Table 2; and The following are the supplementary data related to this article.Supplementary Table 1, Supplementary Table 2.

### 3.3 Resting and exercise echocardiography

Resting and exercise echocardiography main measurements are summarized in Table 3. Exercise echocardiography was the only exercise test performed in eight studies [[10], [11], [12],^14^,^16^,^18^,^21^,^25^], and it was executed along with CPET in three other studies [8,13,15,20].

**Table 1:** Demographic characteristics of the patients.

Study	Region	Period	Overall population, n	Mean follow-up, years	Mean age, years	Men, n(%)	Family history of HCM, n(%)
Efhtimiadis *et al*, 2010 [8] n = 68	Thessaloniki, Greece	2007–2009	68	2	44.8 ± 14.6	45 (67.1)	32(47)
Sorajja *et al*, 2012 [9] n = 182	Rochester (Minnesota), USA	1991–2008	182	4 ± 3.2	53 ± 15	119 (65)	43(24)
Peteiro *et al*, 2012 [10] n = 220	A Coruña, Spain	–	239	4.1 ± 2.6	52 ± 15	145 (61)	76(32)
Reant *et al*, 2014 [11] n = 115	Bordeaux-Pessac, France	2009–2012	115	1.6 ± 0.95	51.9 ± 15.2	76 (66)	59(51)
Desai *et al*, 2014 [12] n = 426	Cleveland (Ohio), USA	1997–2007	426	8.7 ± 3.0	44 ± 14	310 (73)	105(25)
Finocchiaro *et al*, 2015 [13] n = 156	Stanford (California), USA	2007–2012	156	2.25 ± 0.92	51 ± 14	96 (62)	–
Peteiro *et al*, 2015 [14] n = 148	A Coruña, Spain	–	148	7.1 ± 2.7	51 ± 15	97 (65.5)	44(29.7)
Masri *et al*, 2015 [15] n = 1005	Cleveland (Ohio), USA	1997–2012	1005	5.5 ± 4	50 ± 14	643 (64)	201(20)
Feneon *et al*, 2015 [16] n = 126	Rennes and Tours, France	2009–2013	126	2.4 ± 2.0	47.41 ± 15.48	99 (78.1)	42(34.1)
Coats *et al*, 2015 [17] n = 1898	London, United Kingdom	1998–2010	1898	5.6	46 ± 15	1278 (67)	778(42)
Ciampi *et al*, 2016 [18] n = 706	Italy, Spain, Portugal and Serbia	1984–2015	706	4.1	50 ± 16	381 (54)	–
Magri *et al*, 2016 [19] n = 623	Rome, Italy	2007–2015	623	3.7	49 ± 16	429 (69)	–
Moneghetti *et al*, 2017 [20] n = 131	California, USA	2007–2012	131	4.6	52 ± 13	83 (63)	–
Lu *et al*, 2017 [21] n = 536	Baltimore, USA	2005–2015	536	2.1	52 ± 15	359 (67)	110(20.5)
Rigopoulos *et al*, 2018 [22] n = 21	Athenes, Greece	2005–2011	21	2.4 ± 1.08	48.8 ± 13.7	14 (67)	–
Smith *et al*, 2018 [23] n = 589	Michingan, USA	–	589	4.3 ± 3.3	50.7	361 (61.3)	–
Magri *et al*, 2018 [24] n = 681	Italia	2007–2017	681	4.2	48 ± 16	463 (68)	80 (12)
Hamatani *et al*, 2019 [25] n = 42	Osaka, Japan	–	42	2	59 ± 21	14 (33)	–

**Table 2:** Symptoms and co-morbidities.

Study	Angina n (%)	NYHA class > II n (%)	AF n (%)	HT n (%)	Diabetes n (%)	CAD n (%)
Efhtimiadis *et al*, 2010 [8] n = 68	15(22.0)	9(13.2)	14(20.5)	–	–	–
Sorajja *et al*, 2012 [9] n = 182	–	0(0)	24(13)	49(27)	6(3)	7(4)
Peteiro *et al*, 2012 [10] n = 220	90(38)	–	32(13)	–	–	–
Reant *et al*, 2014 [11] n = 115	11(10)	9(8)	16(14)	23(20)	4(3)	4(3)
Desai *et al*, 2014 [12] n = 426	–	0(0)	65(15)	125(32)	21(5)	27(6)
Finocchiaro *et al*, 2015 [13] n = 156	–	22(14)	–	–	–	–
Peteiro *et al*, 2015 [14] n = 148	50(33.8)	–	14(9.4)	–	–	–
Masri *et al*, 2015 [15] n = 1005	150(15)	221(22)	191(19)	422(42)	80(8)	150(15)
Feneon *et al*, 2015 [16] n = 126	14(10.4)	5(4)	–	39(31.0)	–	–
Coats *et al*, 2015 [17] n = 1898	757(41)	145(8)	–	–	–	–
Ciampi *et al*, 2016 [18] n = 706	–	47(7)	–	–	–	–
Magri *et al*, 2016 [19] n = 623	–	37(6)	–	–	–	–
Moneghetti *et al*, 2017 [20] n = 131	–	27(21)	24(18)	–	–	–
Lu *et al*, 2017 [21] n = 536	191(35.6)	68(12.7)	81(15.1)	–	–	–
Rigopoulos *et al*, 2018 [22] n = 21	10 (48)	15 (71)	1 (5)	–	–	4 (19)
Smith *et al*, 2018 [23] n = 589	–	NHYA II-IV 272 (46)	–	–	–	–
Magri *et al*, 2018 [24] n = 681	–	37 (5)	–	170 (25)	27 (4)	34 (5)
Hamatani *et al*, 2019 [25] n = 42	–	–	6 (14)	–	–	–

AF: atrial fibrillation; CAD: coronary artery disease; HT: hypertension; NYHA: New York Heart Association functional class.

**Table 3:** Rest and exercise echocardiographic findings.

Study	MWT mm	Resting LVEF, %	Exercise LVEF, %	LAD mm	LAiV ml/m2	LVOT gradient at rest*	LVOT gradient with exercise*	RWMAs at rest, n (%)	RWMAs with exercise, n (%)	MR at rest n (%)	MR with exercise, n (%)	SAM at rest, n (%)	SAM with exercise, n (%)
Efhtimiadis *et al*, 2010 [8] *n* = 68	21.4 ± 6.5	75.0 ± 11.2	–	42 ± 8	–	- (LVOTO at rest, n(%) = 27(39.7))	–	–	–	–	–	–	–
Sorajja *et al*, 2012 [9] n = 182	19.9 ± 5.2	–	–	–	46.6 ± 8.1	46.3 ± 38.5	–	–	–	–	–	–	–
Peteiro *et al*, 2012 [10] n = 220	20 ± 5	69 ± 9	73 ± 10	44 ± 7	–	25 ± 32 (LVOTO at rest, n (%) = 60(25))	50 ± 54 >30 mmHg, n (%) = 103(43); >50 mmHg, n (%) = 83(35)	5(2)	19(7.9)	40(17)	67(28)	–	–
Reant *et al*, 2014 [11] n = 115	21.3 ± 4.8	71.0 ± 6.9	71.2 ± 6.8	–	35.1 ± 18.1	30.7 ± 33.5 (LVOTO at rest, n (%) = 42(37)	43.5 ± 44.5 (Exercise LVOT gradient > 50 mmHg, n (%) = 34(30))	–	–	51(45)	49(43)	30(26)	–
Desai *et al*, 2014 [12] n = 426	20 ± 5	61 ± 5	–	42 ± 8	–	28 ± 32	62 ± 47	–	–	381(89)	381(89)	105(25)	233(55)
Finocchiaro *et al*, 2015 [13] n = 156	17 ± 5	67 ± 11	–	–	44 ± 19	- (LVOTO at rest, n(%) = 40(27))	- (Exercise LVOT gradient > 50 mmHg, n (%) = 54(35))	–	–	15(10)	–	–	–
Peteiro *et al*, 2015 [14] n = 148	20 ± 5	71 ± 9	73 ± 10	44 ± 6	–	10(9–25) (LVOTO at rest, n (%) = 35(24))	26(10−100) (Exercise LVOT gradient > 30 mmHg, n (%) = 66(45))	3(2)	13(9)	23(15.5)	36(24)	–	–
Masri *et al*, 2015 [15] n = 1005	21 ± 5	62 ± 6	–	44 ± 24	–	41 ± 39	92 ± 51	–	–	958(95.4)	934(93)	391(39)	763(76)
Feneon *et al*, 2015 [16] n = 126	–	66 ± 8	72 ± 15	52 ± 8	25[14]	7[8] (LVOT gradient at rest > 50 mmHg, n (%) = 11(8.7))	12[12] (Exercise LVOT gradient > 50 mmHg, n (%) = 16(12.7))	–	–	–	22(20.9)	–	–
Coats *et al*, 2015 [17] n = 1898	19 ± 5	65 ± 11	–	44 ± 8	–	–	–	–	–	221(12)	–	–	–
Ciampi *et al*, 2016 [18] n = 706	20 ± 5	66 ± 9	–	–	–	- (LVOTO at rest, n(%) = 107(15))	- (Exercise LVOTO (LVOT gradient > 50 mmHg, n(%) = 116(20)	–	35(6)	92(13)	–	–	–
Magri *et al*, 2016 [19] n = 623	20 ± 5	63 ± 7	–	42 ± 7	–	12(7–34)	–	–	–	–	–	–	–
Moneghetti *et al*, 2017 [20] n = 131	–	64 ± 9	–	–	44 ± 17	- (LVOTO at rest n (%) = 41(31)	57 ± 52	–	–	48(37)	–	–	–
Lu *et al,* 2017 [21] n = 536	22 ± 6	65.3 ± 9	–	43 ± 7	–	38.1 ± 17.2	80 ± 39.3	–	–	–	–	–	–
Rigopoulos *et al*, 2018 [22] n = 21	19.7 ± 4.5	–	–	49 ± 6	–	67.1 ± 25.8	–	.	.	15(71)	–	–	–
Smith *et al*, 2018 [23] n = 589	18.8 ± 4.9	–	–	–	–	38.1 (LVOTO at rest, n (%) = 339 (57.5))	.	.	.	–	–	–	–
Magri *et al*, 2018 [24] n = 681	20 ± 5	63 ± 6	–	42 ± 7	–	12 (6–35) (LVOTO at rest, n(%) = 221 (32))	–	–	–	–	–	–	–
Hamatani *et al*, 2019 [25] n = 42	18 ± 4	61 ± 6	–	42 ± 8	51 ± 17	10 (5–18)	29 (12–62)	–	–	7 (17)	15 (36)	–	–

LAD: left atrial diameter; LAiV: left atrial indexed volume; LVEF: left ventricular ejection fraction; LVOT: left ventricular outflow-tract; LVOTO: LVOT obstruction; MWT: maximal wall thickness; RWMAs: regional wall motion abnormalities; MR: mitral regurgitation; SAM: systolic anterior motion of the mitral valve; SD: standard deviation. *mean ± SD or median (interquartile range).

For resting echocardiography, maximal wall thickness was between 17 ± 5 and 22 ± 6 mm. LVEF was preserved (>50%) in all studies, according to the current reference values [29], and in general was minimally (slight increase between 0 and 6% in 4 studies) or not changed with exercise. Mean LVOT pressure gradient at rest was >30 mmHg in six of the studies [9,11,15,[21], [22], [23]], which is diagnostic of LVOTO [2], and during exercise it was >50 mmHg in five studies [10,12,15,20,21]. When evaluated both at rest and during exercise it worsened by, at least, 5 mmHg. Regional wall motion abnormalities (WMAs) appeared *de novo* or worsened with exercise in the three studies evaluating this parameter; this is around four times more patients, when compared with the same parameter evaluated on resting echocardiography [10,14,18]. Not all studies that evaluated mitral regurgitation (MR) at rest repeated this evaluation in exercise echocardiography and one study [16] only reported it with exercise. MR at rest was present in between 10% and 95.4% of patients in all studies evaluating this parameter and between 24% and 93% of patients had MR during exercise, since it appeared *de novo* in some cases. In others, MR progressed to higher degrees of severity. Between 25% and 49.1% of patients had SAM at rest, defined as any contact of the leaflet with the septum during systole [11,12,15]. In the studies that also evaluated this parameter during exercise it was shown that it generally appeared in patients who did not have it at rest and worsened in the ones who had [12,15].

### 3.4 Cardiopulmonary exercise testing

Cardiopulmonary exercise testing was performed in isolation in seven studies [8,9,17,19,[21], [22], [23], [24]] and together with exercise echocardiography in three [13,15,20]. Main measurements regarding CPET are summarized in Table 4.

**Table 4:** Cardiopulmonary exercise testing findings.

Study	Peak VO2, ml/kg/min	% of Predicted Peak VO2 or % of patients with reduced peak VO2	VE/VCO2	Anaerobic threshold, ml/kg/min	RER
Efhtimiadis *et al*, 2010 [8] n = 68	28.3 ± 8.7	79.1 ± 27.5	27.3 ± 4.6	21.8 ± 6.9	1.16 ± 0.11
Sorajja *et al*, 2012 [9] n = 182	22.7 ± 7.6	75 ± 21	31.9 ± 4.7	–	1.13 ± 0.12
Finocchiaro *et al*, 2015 [13] n = 156	26 ± 10	–	29.3 ± 6.7	–	–
Masri *et al*, 2015 [15] n = 1005	21 ± 6	(Peak VO2 < 50%, n(%) = 150(15))	20 ± 17	–	1.09 ± 0.17
Coats *et al*, 2015 [17] n = 1898	22.0 ± 9.1	67 ± 21	32.6 ± 7.3	11.7 ± 4.2	1.10 ± 0.11
Magri *et al*, 2016 [19] n = 623	21 ± 7	71 ± 20	29 ± 6	–	–
Moneghetti *et al*, 2017 [20] n = 131	26 ± 11	(Peak VO2 < 80%, n(%) = 53(40))	(VE/VCO2 > 32, n(%) = 26(20))	–	1.10 ± 0.09
Rigopoulos *et al*, 2018 [22] n = 21	17.7 ± 4.8	66.4 ± 18.7	31.6 ± 5.6	11.1 ± 3.6	1.1 ± 0.1
Smith *et al*, 2018 [23] n = 589	–	76.5 ± 22.4	–	–	1.13 ± 0.1
Magri *et al*, 2018 [24] n = 681	21.6 ± 6.9	72 ± 20	–	–	–

Peak VO2: peak oxygen consumption; VE / VCO2: ventilation/carbon dioxide output; RER: respiratory exchange ratio.

Among the evaluated parameters in CPET, peak VO2 and VE/VCO2 were the ones that were consistently analyzed in all the studies that performed CPET, and also those that demonstrated a higher correlation with outcomes, as discussed below. Mean peak VO2 varied between 17.7 ± 6 and 28.3 ± 8.7 and mean VE/VCO2 varied between 20 ± 17 and 32.6 ± 7.3. In a study that compared HCM patients to a control population [20], it was shown that in the HCM population peak VO2 was lower and VE/VCO2 slope was higher than in controls. Percentage of predicted peak VO2 (generally based on age and sex) was <80% on average.

### 3.5 Other resting and exercise parameters

Other resting and exercise parameters are summarized in Table 5.

**Table 5:** Other resting and exercise parameters.

Study	Heart rate at rest, bpm	Peak heart rate, Bpm	HRR, bpm or % of patients with abnormal HRR	Systolic BP at rest, mmHg	Peak systolic BP, mmHg	ABPR, n (%)
Efhtimiadis *et al*, 2010 [8] n = 68	74.9 ± 14.9	150.5 ± 24.5	–	123.9 ± 16.9	162.1 ± 29.9	–
Sorajja *et al*, 2012 [9] n = 182	71 ± 14	135 ± 27	–	120 ± 18	153 ± 38	–
Peteiro *et al,* 2012 [10] n = 220	–	144 ± 28	–	–	161 ± 31	99(41)
Reant *et al,* 2014 [11] n = 115	67 ± 11	127 ± 23	–	132 ± 20	168 ± 31	17(15)
Desai *et al,* 2014 [12] n = 426	–	150 ± 26	31 ± 14	–	168 ± 35	5(1.2)
Finocchiaro *et al*, 2015 [13] n = 156	68 ± 13	139 ± 27	–	118 ± 20	222 ± 78	–
Peteiro *et al*, 2015 [14] n = 148	–	147 ± 27	–	–	160(140–180)	50(34)
Masri *et al*, 2015 [15] n = 1005	–	136 ± 26	- (Abnormal HRR, n(%) = 231(23))	–	–	10(1)
Coats *et al*, 2015 [17] n = 1898	71 ± 15	138 ± 30	–	126 ± 21	71 ± 35	406(21)
Ciampi *et al*, 2016 [18] n = 706	–	–	–	–	–	141(23)
Magri *et al,* 2016 [19] n = 623	–	- (% of predicted = 81 ± 14)	–	–	- (difference between systolic BP with exercise and at rest,mmHg = 44 ± 24)	–
Moneghetti *et al*, 2017 [20] n = 131	67 ± 12	138 ± 29	–	119 ± 19	158 ± 27	5(4)
Rigopoulos *et al*, 2018 [22] n = 21	–	–	.	–	–	9 (43)
Smith *et al*, 2018 [23] n = 589	–	–	–	–	152.2 ± 24	192 (33)
Magri *et al*, 2018 [24] n = 681	74 ± 15	131 ± 26	–	–	–	–
Hamatani *et al*, 2019 [25] n = 42	64 ± 10	112 ± 22	–	128 ± 23	167 ± 22	–

ABPR: abnormal blood pressure response; HRR: Heart Rate Recovery; BP: blood pressure; bpm: beats per minute.

Mean HRR was, in the two studies [8,12] that reported this parameter, over 12 beats per minute (bpm), the threshold below which is considered abnormal, although this parameter demonstrated to be relevant in terms of prognosis, as discussed below. Abnormal BP response, which has been considered a risk factor for SCD, occurred in between 1% and 43% of the patients.

### 3.6 Follow-up and outcomes

Adverse events during follow-up were previously defined (see methods). The main predictors of worse outcomes were abnormal HRR [12], chronotropic incompetence [8,24], abnormal blood pressure response [23], AF at rest [12], low global longitudinal strain on echocardiogram [11], high left atrium volume [13], exercise WMAs [10,14], higher VE/VCO2 slope, lower peak VO2 [13,17] and pulmonary hypertension (PH) (defined as a mean pulmonary arterial pressure ≥ 25 mmHg at rest /exercise-induced pulmonary hypertension (EIPH) defined as pulmonary artery systolic pressure ≥ 60 mmHg during exercise [25]). The primary endpoints of most studies were combined and highly variable between the different publications. Table 6 summarizes the outcome data.

**Table 6:** Predictors of outcomes.

Study	Composite endpoint (CE)	Predictor of outcome
Desai *et al*, 2014 [12] *n* = 426	Death, appropriate ICD discharges, resuscitated sudden death, and admission for CHF	• Abnormal heart rate recovery at 1 min in recovery: HR 0.89 (0.82–0.97), *p* 0.007 - > 35 *vs* 8% (p < 0.001) meeting the CE • Atrial fibrillation: HR 2.73 (1.30–5.74), *p* 0.007 - >29 *vs* 10% (*p* < 0.001) meeting the CE
Efhtimiadis *et al*, 2010 [8] n = 68	Cardiovascular death, ventricular tachycardia/ventricular fibrillation, and ICD discharge	• Chronotropic incompetence group - low heart rate reserve (53.0 ± 4.0 bpm) - > 15 *vs* 0% (p 0.05) meeting the CE
Magri *et al*, 2018 [24] n = 681	HF endpoint (death from HF, cardiac transplantation, progression to NYHA class III–IV,admission for CHF, and septal reduction procedure)	• pHR equal to 70% as the best cut-off value in predicting the HF end-point HR 2.9 (*p <* 0.001) (sensitivity: 62%; specificity: 72%; AUC: 0.68)
	Arrhythmia endpoint (SCD, aborted SCD and appropriate ICD shock)	• pHR% equal to 65% as the best cut-off value in predicting the SCD end-point (sensitivity: 40%; specificity: 80%; AUC: 0.56)
Smith *et al*, 2018 [23] n = 589	HF endpoint (first heart failure hospitalization)	• ABPR at baseline testing were more likely to have a subsequent heart failure hospitalization (*p* = 0.002). The presence or absence of LVOTO did not alter this association.
	Arrhythmia endpoint (SCD, aborted SCD and appropriate ICD shock)	• The adverse arrhythmia endpoint was not associated with ABPR (*p* = 0.270)
Reant *et al,* 2014 [11] n = 115	Death related to HCM (SCD, death from HF, or stroke related to AF), SVT, appropriate cardiac shock or resuscitated cardiac arrest, and progression NYHA III or IV	• Global longitudinal strain <15%, HR 3.29 (*P* = 0.028); (sensitivity of 67%, specificity of 77%, and AUC 0.754)
Peteiro *et al,* 2012 [10] n = 220	Cardiac death, cardiac transplantation, appropriate ICD shock, SVT, stroke related to AF, myocardial infarction, and HF requiring hospitalization	• Exercise WMAs were more frequent in patients who developed hard events (31.5% *vs* 5.9%, P < 0.001)
Peteiro *et al*, 2015 [14] n = 148	Cardiac death, cardiac transplantation, appropriate ICD shock, SVT, stroke related to AF, myocardial infarction, and HF requiring hospitalization	• Exercise WMAs were more frequent in patients who developed hard events (57 *vs.* 6%, P < 0.001)
Finocchiaro *et al*, 2015 [13] n = 156	Overall mortality, heart transplantation, and functional deterioration leading to hospitalization for septal reduction	• Peak VO < 80% of predicted (HR: 4.11; 95% confidence in- terval [CI]: 1.46 to 11.59; p 0.008) • VE/VCO slope > 34 (HR: 3.14; 95% CI: 1.26 to 7.87; p 0.014) • Left atrial volume > 40 ml/m2 (HR: 3.32; 95% CI: 1.08 to 10.16; p 0.036)
Coats *et al*, 2015 [17] n = 1898	All-cause mortality or cardiac transplantation.	• Peak V̇O2 (adjusted HR 0.85, 95% CI 0.77–0.92, P < 0.001) and VĖ V̇CO2 slope (adjusted HR 0.85, 95% CI 0.77–0.92, *P* < 0.001) were both independent predictors of the CE
Hamatani *et al*, 2019 [25] n = 42	SVT, hospitalization due to HF, and AF events (new-onset AF or hospitalization due to AF such as cardioversion)	• EIPH had a significantly higher incidence of HCM-related morbidity than those without EIPH (log-rank; *P* = 0.01)

ICD: implantable cardiac defibrillator; CHF: chronic heart failure, pHR: maximum age-predicted heart rate; SCD: sudden cardiac death; HF: heart failure; AUC: area under the curve; AF: atrial fibrillation; SVT: sustained ventricular tachycardia; WMA: wall motion abnormalities; EIPH: exercise-induced pulmonary hypertension.

### 3.7 Limitations of the included studies

The limitations were common to the majority of the studies and included studies being performed by only one centre [16], mostly referral centres for HCM, which means that the sample might not have been representative of the overall HCM population [8,12,13]. Additional selection biases were present, such as only including patients who were able to undergo exercise echocardiography [12] and exclusion of patients in NYHA class IV and with a prior LVEF<50%. It was also noted either a tendency to include more symptomatic patients, because of clinical indication, in some studies [8,13,17] or, in one study, not including more symptomatic patients, since they were referred to surgery before undergoing stress testing [14]. Another limitation of some of the included articles was a small sample size [8,22,25].

In some of the studies, the patients did not withdraw medications (because it was considered unethical), such as beta-blockers and calcium channel blockers, which obviously influenced the hemodynamic response to exercise, diminishing the accuracy of defining an abnormal BP response, an abnormal chronotropic response and blunting the exercise-induced LVOT gradient [11,16].

Many studies were limited due to having a short follow-up period [11,13,21,25] and/or a reduced number of events [11,13,14,16].

Finally, some of the parameters were not uniformly assessed and hence not reported [12], which related to the fact that exercise echocardiography protocols are not standardized in HCM (some are performed in treadmill and others in semi-supine position, and the latter tends to require lower workload). Therefore, it might be challenging to compare different works.

Some of the studies were performed by the same authors and at the same centre, so there might be some overlap of study populations.

In general, the heterogeneity of the methods, reported parameters and outcomes precluded a meta-analysis.

A Downs and Black checklist is provided in supplementary material.

## 4 Discussion

We hereby present an up to date systematic review of contemporary studies evaluating the prognostic value of exercise testing in HCM and report a group of parameters, obtained from either exercise echocardiography or cardiopulmonary exercise testing, that revealed to be predictors of worse outcomes.

**Fig. 2:**
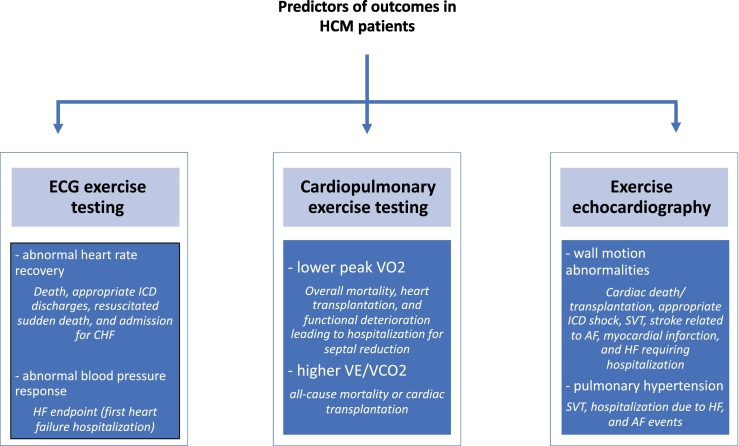
Summary of the main findings of the systematic review. Legend: AF: atrial fibrillation, HF: heart failure, HCM: hypertrophic cardiomyopathy, ICD: implantable cardioverter-defibrillator, SVT: supraventricular tachycardia.

These parameters are summarized in Fig. 2.

Exercise testing, either echocardiographic or cardiopulmonary, allows the evaluation of diverse clinically relevant parameters in the assessment of HCM patients. The role of exercise testing is well defined for symptom evaluation and management. However, its utility in the prediction of outcomes, in order to obtain a more accurate risk stratification and improve the prognostic models, is less well established.

The relevance of performing exercise echocardiography to study LVOTO during exercise was firstly reported in 2006 [30] but only few groups have investigated the prognostic implications. The data correlating peak VO2 and other parameters obtained from cardiopulmonary exercise testing and prognosis have also been scarce.

As expected, some of the parameters evaluated both at rest and with exercise echocardiography in the included studies, including SAM, LVOTO and MR, appeared *de novo* or worsened with exercise, due to the cardiac response to stress.

Correlation of exercise-evaluated parameters with events was the main aim of this systematic review. One of these parameters was an abnormal HRR [8,12], possibly explained by a blunted vagal reactivation in HCM patients [8], and might identify patients with a higher risk of death, malignant arrhythmias and HF progression.

Abnormal BP response to exercise [11] has been previously considered a risk factor for SCD, although not included in the risk score evaluation from the latest ESC guidelines [1]. It was correlated with worse outcomes regarding heart failure hospitalization and with no increase in sudden cardiac death; this association was independent of LVOTO [28]. Abnormal BP response to exercise seemed to be a clinical indicator of a lack of cardiac reserve, rather than an independent risk factor for SCD.

Exercise echocardiogram is also relevant to identify important subgroups of patients without gradients at rest (or with Valsalva) who nevertheless developed hemodynamically significant LV outflow obstruction only with exercise, including some with severe gradients, >50 mmHg. Indeed, a substantial proportion of these patients have limiting heart failure symptoms; therefore, identification of latent, exercise-triggered obstruction not only defines the probable mechanism for such symptoms but in many cases also reveals options for their relief with surgical or interventional septal reduction therapies [30].

AF in patients with HCM is considered to be a risk factor for cardiovascular death, heart failure, and stroke [31,32]. However there are no data regarding the prognostic impact of AF induced by exercise.

Regional exercise WMAs [10], probably explained by myocardial ischemia, were associated with a worse prognosis for HCM patients [14,33], having incremental prognostic value over clinical and resting echocardiographic variables.

Lower peak VO2 (as well as lower percentage of predicted peak VO2) and higher VE/VCO2 slope, that reflects ventilatory inefficiency, are parameters of exercise intolerance and associated with a worse prognosis [13,17,20]. However, two of the studies [13,17] concluded that these were only related with some of the outcomes, namely heart failure and heart transplantation, and not with sudden cardiac death, probably because mechanisms for ventricular arrhythmia and loss of contractile function are different, suggesting the importance of defining prediction parameters for specific outcomes. The largest study [17] concluded that CPET was useful for the risk stratification of patients with both obstructive and non-obstructive forms of the disease. Lower anaerobic threshold was not as much predictive of worse outcomes as peak VO2 and VE/VCO2.

Exercise capacity reflected as NYHA class is part of the criteria used to refer a patient to invasive septal reduction therapy, but a specific VO2 value/threshold is not currently used in this regard [1]. Recently Alashi *et al* [34] suggested that earlier myomectomy by surgery guided by VO2 had better long term survival, raising a new topic for discussion.

PH and/or EIPH appear to be the consequence of the increase of LV filling pressure secondary to diastolic dysfunction, LV obstruction, or mitral regurgitation, which could promote the occurrence of ventricular tachycardia [24]. Moreover, increased LV filling pressure results in atrial overload and remodeling, which might promote the occurrence of HF and AF. Indeed, PH in HCM patients was significantly associated with increased morbidity and EIPH also showed a significant association with HCM-related morbidity, perhaps because EIPH represents the downstream effect of the hemodynamic derangements that increase LV filling pressure during exercise [25].

Some of the assessed parameters were not consistently considered predictors of outcomes by all the studies. For example, indirect indices of diastolic dysfunction, such as LA diameter, were considered a predictor in two studies [13,16], but not in another one [10].

Severity and worsening of LVOTO (*i.e.* higher LVOT gradient at peak exercise) was predictive of outcomes in two studies [9,16], as well as in previous work [30,6], and not associated with events in two other [12,14]. One of the studies pointed out that peak LVOT gradient ≥50 mmHg was more predictive of outcomes than rest LVOT gradient ≥30 mmHg and that peak LVOT gradient was a better predictor than post- exercise measurement [11]. There is still conflicting evidence regarding the prognostic impact of exercise-induced LVOTO in HCM.

MR has been described in patients with HCM since the first reports of the disease and it is commonly associated with LVOTO and SAM. Since it is a dynamic phenomenon, it is important to evaluate this parameter during exercise. Exercise-induced MR was associated with adverse cardiovascular events in two studies [11,16], although in one of them [16] the result was not considered significant. As such, the increase in the degree of MR and its appearance *de novo* with exercise also seems to be of prognostic relevance.

Chronotropic incompetence (*i.e.* a blunted increase in heart rate during exercise, defined as a maximal HR during exercise that is less than 65–80% of the predicted value) is a predictor of clinical outcome in coronary artery disease, congenital heart disease and healthy populations [[35], [36], [37]]. In HCM, it is possibly explained by autonomic dysfunction, sino-atrial electrophysiological remodeling, altered beta-receptors function and density as well as impaired intracellular calcium signaling and was considered an independent predictor of exercise intolerance in one study [8], and an independent predictor of heart-failure related events in another study [24] – defined as a single exercise derived parameter.

In supplementary Table 3 we summarize all the current recommendations regarding the various forms of exercise stress testing in HCM, comparing 2014 ESC guidelines and 2020 AHA/ACC guidelines [1,3].

## 5 Conclusion

Data derived from exercise, which can be assessed with exercise echocardiography and/or cardiopulmonary exercise testing, is able to objectively evaluate functional status but also to risk stratify HCM patients, refining prognostic assessment. Further investigation in this area is warranted, namely larger, multi- center studies with longer follow-up and standardized stress protocols to evaluate whether exercise testing adds independent value to the current risk stratification strategies in HCM.

## Funding

LRL is funded by a 10.13039/501100000265Medical Research Council (MRC) UK Clinical Academic Research Partnership (CARP) award.

## Declaration of Competing Interest

None declared.

The following are the supplementary data related to this article.Supplementary Table 1 Risk factors for sudden cardiac death. Supplementary Table 1

Supplementary Table 2 Medications and implanted devices. Supplementary Table 2

Supplementary Table 3 Clinical impact and role of different types of exercise testing (comparison of 2014 ESC guidelines and 2020 AHA/ACC guidelines. Supplementary Table 3

Supplementary Table 4 Downs and Black checklist for non-randomized studies. Supplementary Table 4

Supplementary material 1 Supplementary methods. Supplementary material 1

Supplementary data to this article can be found online at https://doi.org/10.1016/j.ijcard.2021.06.051.

## References

[1] Elliott P.M., Anastasakis A., Borger M., Borggrefe M., Cecchi F., Charron P., Hagege A.A., Lafont A., Limongelli G., Mahrholdt H., McKenna W.J., Mogensen J., Nihoyannopoulos P., Nistri S., Pieper P.G., Pieske B., Rapezzi C., Rutten F.H., Tillmanns C., Watkins H. (2014). 2014 ESC guidelines on diagnosis and management of hypertrophic cardiomyopathy. Eur. Heart J..

[2] Gersh B.J., Maron B.J., Bonow R.O., Dearani J.A., Fifer M.A., Link M.S., Naidu S.S., Nishimura R.A., Ommen S.R., Rakowski H., Seidman C.E., Towbin J.A., Udelson J.E., Yancy C.W. (2011). ACCF/AHA guideline for the diagnosis and treatment of hypertrophic cardiomyopathy. Circulation.

[3] Ommen S., Mital S., Burke M., Day S., Deswal A., Elliott P., Evanovich L., Hung J., Joglar J. (2020). AHA/ACC guideline for the diagnosis and treatment of patients with hypertrophic cardiomyopathy. Circulation.

[4] Binder R., Wonisch M., Corra U., Cohen-Solal A., Vanhees L., Saner H., Schmid J.P. (2008). Methodological approach to the first and second lactate threshold in incremental cardiopulmonary exercise testing. Eur. J. Cardiovasc. Prev. Rehabil..

[5] Cavigli L., Olivotto I., Fattirolli F., Mochi N., Favilli S., Mondillo S., Bonifazi M., D’Ascenzi F. (2020). Prescribing, dosing and titrating exercise in patients with hypertrophic cardiomyopathy for prevention of comorbidities: ready for prime time.. Eur J Prev Card.

[6] Maron M.S., Olivotto I., Betocchi S., Casey S.A., Lesser J.R., Losi M.A., Cecchi F., Maron B.J. (2003). Effect of left ventricular outflow tract obstruction on clinical outcome in hypertrophic cardiomyopathy. N. Engl. J. Med..

[7] Moher D., Liberati A., Tetzlaff J., Altman D.G., The PRISMA Group (2009). Preferred Reporting Items for Systematic Reviews and Meta-Analyses: The PRISMA Statement. BMJ.

[8] Efthimiadis G.K., Giannakoulas G., Parcharidou D.G., Pagourelias E.D., Kouidi E.J., Spanos G., Kamperidis V., Gavrielides S., Karvounis H., Styliadis I., Parcharidis G.E. (2011). Chronotropic incompetence and its relation to exercise intolerance in hypertrophic cardiomyopathy. Int. J. Cardiol..

[9] Sorajja P., Allison T., Hayes C., Nishimura R.A., Lam C.S.P., Ommen S.R. (2012). Prognostic utility of metabolic exercise testing in minimally symptomatic patients with obstructive hypertrophic cardiomyopathy. Am. J. Cardiol..

[10] Peteiro J., Bouzas-mosquera A., Fernandez X., Moserrat L., Pazos P., Estevez-Loureiro R., Castro-Beiras A. (2012). Prognostic value of exercise echocardiography in patients with hypertrophic cardiomyopathy. J. Am. Soc. Echocardiogr..

[11] Reant P., Reynaud A., Pillois X., Dijos M., Arsac F., Touche C., Landelle M., Rooryck C., Roudaut R., Lafitte S. (2015). Comparison of resting and exercise echocardiographic parameters as indicators of outcomes in hypertrophic cardiomyopathy. J. Am. Soc. Echocardiogr..

[12] Desay M.Y., Bhonsale A., Patel P., Naji P., Smedira N.G., Thamilarasan M., Lytle B.W., Lever H.M. (2014). Exercise echocardiography in asymptomatic HCM. JACC. – Cardiovasc. Imaging..

[13] Finocchiaro G., Haddad F., Knowles J.E., Caleshu C., Pavlovic A., Homburger J., Shmargad Y., Sinagra G., Magavern E., Wong M., Perez M., Schnittger I., Myers J., Groelicher V., Ashley E.A. (2015). Cardiopulmonary responses and prognosis in hypertrophic cardiomyopathy: a potential role for comprehensive noninvasive hemodynamic assessment. JACC: Heart Fail..

[14] Peteiro J., Fernandez X., Bouzas-Mosquera A., Moserrat L., Mendez C., Rodriguez-Garcia E., Soler R., Couto D., Castro-Beiras A. (2015). Exercise echocardiography and cardiac magnetic resonance imaging to predict outcome in patients with hypertrophic cardiomyopathy. Eur. Heart J..

[15] Masri A., Pierson L.M., Smedira N.G., Agarwal S., Lytle B.W., Naji P., Thamilarasan M., Lever H.M., Cho L.S., Desay M.Y. (2015). Predictors of long-term outcomes in patients with hypertrophic cardiomyopathy undergoing cardiopulmonary stress testing and echocardiography. Am. Heart J..

[16] Feneon D., Schnell F., Galli E., Bernard A., Mabo P., Daubert J.C., Leclerq C., Carre F., Donal E. (2016). Impact of exercise-induced mitral regurgitation on hypertrophic cardiomyopathy outcomes. Eur. Heart J. – Cardiovasc. Imag..

[17] Coats C.J., Rantell K., Bartnik A., Patel A., Mist B., McKenna W., Elliott P.M. (2015). Cardiopulmonary exercise testing and prognosis in hypertrophic cardiomyopathy. Circulation.

[18] Ciampi Q., Olivotto I., Gardini C., Mori F., Peteiro J., Moserrat L., Fernandez X., Cortigiani L., Rigo F., Lopes L.R., Cruz I., Cotrim C., Losi M., Betocchi S., Beleslin B., Tesic M., Dikic A.D., Lazzeroni E., Lazzeroni D., Sicari R., Picano E. (2016). Prognostic role of stress echocardiography in hypertrophic cardiomyopathy: the international stress echo registry. Int. J. Cardiol..

[19] Magrì D., Limongelli G., Re F., Agostoni P., Zachara E., Correale M., Mastromarino V., Santolamazza C., Casenghi M., Pacileo Valente F., Musumeci B., Maruotti A., Volpe M., Autore C. (2016). Cardiopulmonary exercise test and sudden cardiac death risk in hypertrophic cardiomyopathy. Heart.

[20] Moneghetti K.J., Stolfo D., Christle J.W., Kobayashi Y., Finocchiaro G., Sinagra G., Myers J., Ashley E.A., Haddad F., Wheeler M.T. (2017). Value of strain imaging and maximal oxygen consumption in patients with hypertrophic cardiomyopathy. Am. J. Cardiol..

[21] Lu D., Hailesealassie B., Ventoulis I., Liu H., Liang H.Y., Nowbar A., Pozios I., Canepa M., Cresswell K., Luo H.C., Abraham M.R., Abraham T.P. (2017). Impact of peak provoked left ventricular outflow tract gradients on clinical outcomes in hypertrophic cardiomyopathy. Int. J. Cardiol..

[22] Rigopoulos A., Panou F., Sakadakis E., Frogoudaki A., Papadopoulou K., Triantafyllidi H., Ali M., Iliodromitis E., Rizos I., Noutsias M. (2018). Cardiopulmonary exercise test parameters at three months after alcohol septal ablation in hypertrophic obstructive cardiomyopathy are associated with late clinical outcome. Heart, Lung Circul..

[23] Smith E., Tome J., Mcgrath R., Kumar S., Concannon M., Day S.M., Saberi S., Helms A.S. (2018). Exercise hemodynamics in hypertrophic cardiomyopathy identify risk of incident heart failure but not ventricular arrhythmias or sudden cardiac death. Int. J. Cardiol..

[24] Magri D., Agostoni P., Sinagra G., Re F., Correale M., Limongelli G., Zachara E., Mastromarino V., Santolamazza C., Casenghi M., Pacileo G., Valente F., Morosin M., Musumeci B., PAgannone E., Maroutti A., Uguccioni M., Volpe M., Autore C. (2017). Clinical and prognostic impact of chronotropic incompetence in patients with hypertrophic cardiomyopathy. Int. J. Cardiol..

[25] Hamatani Y., Amaki M., Yonezawa R., Yanagi Y., Jo Y., Amano M., Okada A., Takahama H., Hasegawa T., Kanzaki H., Yasuda S., Izumi C. (2019). Prevalence, determinants, and prognostic significance of exercise-induced pulmonary hypertension in patients with hypertrophic cardiomyopathy. Int. J. Cardiovasc. Imag..

[26] Cole C.R., Blackstone E.H., Pashkow F.J., Snader C.E., Lauer M.S. (1999). Heart-rate recovery immediately after exercise as a predictor of mortality. N. Engl. J. Med..

[27] Olivotto I., Maron B.J., Montereggi A., Mazzuoli F., Dolara A., Cecchi F. (1999). Prognostic value of systemic blood pressure response during exercise in a community-based patient population with hypertrophic cardiomyopathy. J. Am. Coll. Cardiol..

[28] O’Mahony C., Jichi F., Pavlou M., Monserrat L., Anastasakis A., Rapezzi C., Biagini E., Gimeno J., Limongelli G., McKenna W., Omar R., Elliott P. (2014). A novel clinical risk prediction model for sudden cardiac death in hypertrophic cardiomyopathy (HCM risk-SCD). Eur. Heart J..

[29] Lang R.M., Badano L.P., Mor-Avi V., Afilalo J., Armstrong A., Ernande L., Flachskampf F.A., Foster E., Goldstein S.A., Kuznetsova T., Lancellotti P., Muraru D., Picard M.H., Rietzschel E.R., Rudski L., Spencer K.T., Tsang W., Voigt J.U. (2015). Recommendations for cardiac chamber quantification by echocardiography in adults: an update from the American Society of Echocardiography and the European Association of Cardiovascular Imaging. Eur. Heart J. – Cardiovasc. Imag..

[30] Maron M.S., Olivotto I., Zenovich A.G., Link M.S., Pandian N.G., Kuvin J.T., Nistri S., Cecchi F., Udelson J.E., Maron B.J. (2006). Hypertrophic cardiomyopathy is predominantly a disease of left ventricular outflow tract obstruction. Circulation.

[31] Azarbal F., Singh M., Finocchiaro G., Le V.V., Schnittger I., Wang P., Myers J., Ashley E., Perez M. (2014). Exercise capacity and paroxysmal atrial fibrillation in patients with hypertrophic cardiomyopathy. Heart..

[32] Siontis K.C., GEske J.B., Ong K., Nishimura R.A., Ommen S.R., Gersh B.J. (2014). Atrial fibrillation in hypertrophic cardiomyopathy: prevalence, clinical correlations, and mortality in a large high-risk population. J. Am. Heart Assoc..

[33] Okeie K., Shimizu M., Yoshio H., Ino H., Yamaguchi M., Matsuyama T., Yasuda T., Taki K., Mabuchi H. (2000). Left ventricular systolic dysfunction during exercise and dobutamine stress in patients with hypertrophic cardiomyopathy. J. Am. Coll. Cardiol..

[34] Alashi A., Smedira N., Hodges K., Popovic Z., Thamilarasan M., Wierup P., Lever H., Desai M. (2021). Outcomes in guideline-based class I indication versus earlier referral for surgical myectomy in hypertrophic obstructive cardiomyopathy. J. Am. Heart Assoc..

[35] Diller G.P., Dimopoulos K., Okonko D., Uebing A., Broberg C.S., Babu-Narayan S., Bayne S., Poole-Wilson P.A., Sutton R., Francis D.P., Gatzoulis M.S. (2006). Heart rate response during exercise predicts survival in adults with congenital heart disease. J. Am. Coll. Cardiol..

[36] Jouven X., Empana J.P., Schwartz P.J., Desnos M., Courbon D., Ducimetière P. (2005). Heart-rate profile during exercise as a predictor of sudden death. N. Engl. J. Med..

[37] Lauer M.S., Francis G.S., Okin P.M., Pashkow F.J., Snader C.E., Marwick T.H. (1999). Impaired chronotropic response to exercise stress testing as a predictor of mortality. J. Am. Med. Assoc..

