# Supplementary material for: Prognostic relevance of exercise testing in hypertrophic cardiomyopathy. A systematic review
Source: Int J Cardiol. 2021 Sep 15;339:83–92. doi: 10.1016/j.ijcard.2021.06.051 (PMC8425182; doi:10.1016/j.ijcard.2021.06.051)
Supplement: The following are the supplementary data related to this article.Supplementary Table 1 — Risk factors for sudden cardiac death. [file mmc1.docx]

Supplementary table 1 – Risk factors for sudden cardiac death

| Study | Syncope  n (%) | Family history of SCD  n (%) | NSVT  n (%) | ABPR to exercise  n (%) | MLVWT ≥30mm  n (%) |
| --- | --- | --- | --- | --- | --- |
| Efhtimiadis *et al*, 2010 (8) n=68 | 10(14.7) | 17(25) | 10(19.6) | 19(27.9) | 8(11.7) |
| Sorajja *et al*, 2012 (9) n=182 | - | 21(12) | - | - | - |
| Peteiro *et al*, 2012 (10) n=220 | 26(11) | - | 35(15) | - | - |
| Reant *et al*, 2014 (11)  n=115 | 29(25) | 44(38) | 21(18) | 17(15) | 13(11) |
| Desai *et al*, 2014 (12) n=426 | 69(16) | 105(25) | - | - | - |
| Finocchiaro *et al*, 2015 (13) n=156 | - | - | - | - | - |
| Peteiro *et al*, 2015 (14) n=148 | 13(8.8) | 15(10) | 20(13.5) | - | - |
| Masri *et al*, 2015 (15) n=1005 | 171(17) | - | - | - | - |
| Feneon *et al*, 2015 (16) n=126 | 16(51.9) | 28(20.7) | 18(10.8) | - | 7(5.2) |
| Coats *et al*, 2015 (17) n=1898 | 216(12) | 321(17) | 531(28) | - | 70(4) |
| Ciampi *et al*, 2016 (18) n=706 | - | - | - | 141(23) | - |
| Magri *et al*, 2016 (19) n=623 | 94(15) | 71(11) | 214(34) | 114(18) | 42(7) |
| Moneghetti *et al*, 2017 (20) n=131 | 34(26) | 36(28) | 40(30) | 5(4) | - |
| Lu *et al*, 2017 (21) n=536 | 104(19.4) | 134(25) | 15(2.8) | - | - |
| Rigopoulos *et al,* 2018 (22) n = 21 | 3 (14) | - | 0 (0) | 9 (43) | - |
| Smith *et al,* 2018 (24) n = 589 | - | - | 73 (12) | 192 (33) | - |
| Magri *et al,* 2018 (25) n = 681 | 96 (14) | 80 (12) | 219 (32) | 111 (16) | 48 (7) |
| Hamatani *et al,* 2019 (26) n = 42 | - | - | 8 (19) | - | - |

ABPR: Abnormal blood pressure response; SCD: sudden cardiac death; MLVWT: maximal left ventricular wall thickness; NSVT: non-sustained ventricular tachycardia.
