# Supplementary material for: Prognostic relevance of exercise testing in hypertrophic cardiomyopathy. A systematic review
Source: Int J Cardiol. 2021 Sep 15;339:83–92. doi: 10.1016/j.ijcard.2021.06.051 (PMC8425182; doi:10.1016/j.ijcard.2021.06.051)
Supplement: Supplementary Table 2 — Medications and implanted devices. [file mmc2.docx]

Supplementary table 2 –Medications and implanted devices

| Study | β-blocker  n(%) | CCB  n(%) | Permanent PM  n(%) | ICD  n (%) |
| --- | --- | --- | --- | --- |
| Efhtimiadis *et al*, 2010 (8) n=68 | 34(50.0) | 2(2.9) | - | - |
| Sorajja *et al*, 2012 (9) n=182 | 89(49) | 41(23) | - | - |
| Peteiro *et al*, 2012 (10) n=220 | 93(39) | 29(12) | - | 2(1) |
| Reant *et al*, 2014 (11) n=115 | - | - | - | - |
| Desai *et al*, 2014 (12) n=426 | 253(60) | 87(20) | 30(7) | 33(8) |
| Finocchiaro *et al*, 2015 (13) n=156 | 82(53) | 38(24) | - | - |
| Peteiro *et al*, 2015 (14) n=148 | 49(33.1) | 19(12.8) | - | 0(0) |
| Masri *et al*, 2015 (15) n=1005 | 773(77) | 291(29) | 100(10) | 110(11) |
| Feneon *et al*, 2015 (16) n=126 | 95(75.4) | 17(13.5) | - | 16(12.7) |
| Coats *et al*, 2015 (17) n=1898 | 970(51) (b-blocker or CCB) | | 88 (5) | 63 (3) |
| Ciampi *et al*, 2016 (18) n=706 | 336(50) (b-blocker or CCB) | | 76(11) | - |
| Magri *et al*, 2016 (19) n=623 | 417(67) (b-blocker or CCB) | | 56(9) | - |
| Moneghetti *et al*, 2017 (20) n=131 | 81(62) | 39(30) | - | 51(39) |
| Lu *et al*, 2017 (21) n=536 | 378(70.5) | 150(28) | - | 47(8.7) |
| Rigopoulos *et al*, 2018 (22) n = 21 | 19 (90) | 6 (29) | - | - |
| Smith *et al*, 2018 (24) n = 589 | 431 (73) | - | - | |
| Magri *et al*, 2018 (25) n = 681 | 449 (66) | 48 (7) | - | 74 (11) |
| Hamatani *et al*, 2019 (26) n = 42 | 27 (64) | 5 (12) | 9 (21) – permanent PM or ICD | |

CCB: calcium channel blocker; ICD: implanted cardioverter defibrillator; PM: pacemaker
