# Supplementary material for: Prognostic relevance of exercise testing in hypertrophic cardiomyopathy. A systematic review
Source: Int J Cardiol. 2021 Sep 15;339:83–92. doi: 10.1016/j.ijcard.2021.06.051 (PMC8425182; doi:10.1016/j.ijcard.2021.06.051)
Supplement: Supplementary Table 3 — Clinical impact and role of different types of exercise testing (comparison of 2014 ESC guidelines and 2020 AHA/ACC guidelines. [file mmc3.docx]

Supplementary table 3 – Clinical impact and role of different types of exercise testing (comparison of 2014 ESC guidelines and 2020 AHA/ACC guidelines

| Exercise testing | 2014 ESC guidelines | 2020 AHA/ACC guidelines |
| --- | --- | --- |
| Stress echocardiography | In symptomatic patients with a resting or provoked peak instantaneous LV outflow tract gradient <50 mm Hg, 2D and Doppler echocardiography during exercise in the standing, sitting or semi-supine position is recommended to detect provocable LVOTO and exercise-induced mitral regurgitation. (I – B) | For symptomatic patients with HCM who do not have resting or provocable outflow tract gradient ≥50 mm Hg on TTE, exercise TTE is recommended for the detection and quantification of dynamic LVOTO (I – B-NR) |
| Stress echocardiography | In asymptomatic patients with a resting or provoked peak instantaneous LV outflow tract gradient <50 mm Hg, 2D and Doppler echocardiography during exercise—in the standing, sitting or semi-supine positions—may be considered when the presence of an LVOT gradient is relevant to lifestyle advice and decisions on medical treatment. (IIb – C) | For asymptomatic patients with HCM who do not have a resting or provocable outflow tract gradient ≥50 mm Hg on standard TTE, exercise TTE is reasonable for the detection and quantification of dynamic LVOTO. (IIa – C-LD) |
| Cardiopulmonary exercise test | Cardiopulmonary exercise testing, with simultaneous measurement of respiratory gases, is recommended in severely symptomatic patients with systolic and/or diastolic LV dysfunction being evaluated for heart transplantation or mechanical support. (I -B) | In patients with nonobstructive HCM and advanced HF (NYHA functional class III to class IV despite GDMT), cardiopulmonary exercise stress testing should be performed to quantify the degree of functional limitation and aid in selection of patients for heart transplantation or mechanical circulatory support. (I – B-NR) |
| Cardiopulmonary exercise test | Irrespective of symptoms, cardiopulmonary exercise testing with simultaneous measurement of respiratory gases (or standard treadmill or bicycle ergometry when unavailable) should be considered to assess the severity and mechanism of exercise intolerance and change in systolic blood pressure. (IIa-B) | - |
| Cardiopulmonary exercise test | Cardiopulmonary exercise testing, with simultaneous measurement of respiratory gases (or standard treadmill or bicycle ergometry when unavailable), should be considered in symptomatic patients undergoing septal alcohol ablation and septal myectomy to determine the severity of exercise limitation. (IIa- B) | - |
| Cardiopulmonary exercise test | Cardiopulmonary exercise testing (when available) may be considered every 2–3 years in clinically stable patients, or every year in patients with progressive symptoms. (IIb – C) | - |
| Exercise stress test | - | In patients with HCM, exercise stress testing is reasonable to determine functional capacity and to provide prognostic information as part of initial evaluation. (IIa – B-NR) |
| Exercise stress test | - | In patients with obstructive HCM who are being considered for SRT and in whom functional capacity or symptom status is uncertain, exercise stress testing may be reasonable. (IIb – C-EO) |
| Exercise stress test | Symptom-limited exercise testing should be considered every 2–3 years in clinically stable patients, or every year in patients with progressive symptoms. (IIa - C) | In patients with HCM in whom functional capacity or symptom status is uncertain, exercise stress testing may be considered every 2 to 3 years (IIb – C-EO) |

TTE: transthoracic echocardiogram, LVOTO: left ventricular outflow obstruction, NR: nonrandomized, LD – limited data, SRT: septal reduction therapy, EO – expert opinion
