# Supplementary material for: Prognostic relevance of exercise testing in hypertrophic cardiomyopathy. A systematic review
Source: Int J Cardiol. 2021 Sep 15;339:83–92. doi: 10.1016/j.ijcard.2021.06.051 (PMC8425182; doi:10.1016/j.ijcard.2021.06.051)
Supplement: Supplementary Table 4 — Downs and Black checklist for non-randomized studies. [file mmc4.docx]

Supplementary table 4 - Downs and Black checklist for non-randomized studies

| Question  No. | Efthimiadis 2011^5^ | Sorajja 2012^6^ | Peteiro 2012^7^ | Reant 2015^8^ | Desay 2014^9^ | Finocchiaro 2015^10^ | Peteiro 2015^11^ | Masri 2015^12^ | Feneon 2016^13^ | Coats 2015^14^ | Ciampi 2016^15^ | Magri 2016^16^ | Moneghetti 2017^17^ | Lu 2017^18^ | Rigopoulos 2018^19^ | Smith 2018^20^ | Magri 2017^21^ | Hamatani 2019^22^ |
| --- | --- | --- | --- | --- | --- | --- | --- | --- | --- | --- | --- | --- | --- | --- | --- | --- | --- | --- |
| 1 | Yes | Yes | Yes | Yes | Yes | Yes | Yes | Yes | Yes | Yes | Yes | Yes | Yes | Yes | Yes | Yes | Yes | Yes |
| 2 | Yes | Yes | Yes | Yes | Yes | Yes | Yes | Yes | Yes | Yes | Yes | Yes | Yes | Yes | Yes | Yes | Yes | Yes |
| 3 | Yes | Yes | Yes | Yes | Yes | Yes | Yes | Yes | Yes | Yes | Yes | Yes | Yes | Yes | Yes | Yes | Yes | Yes |
| 4 | Yes | Yes | Yes | Yes | Yes | Yes | Yes | Yes | Yes | Yes | Yes | Yes | Yes | Yes | Yes | Yes | Yes | Yes |
| 5 | No | Yes | Yes | No | Yes | Yes | Yes | No | Yes | Yes | Yes | Yes | Yes | No | No | No | No | No |
| 6 | Yes | Yes | Yes | Yes | Yes | Yes | Yes | Yes | Yes | Yes | Yes | Yes | Yes | Yes | Yes | Yes | Yes | Yes |
| 7 | Yes | Yes | Yes | Yes | Yes | Yes | Yes | Yes | Yes | Yes | Yes | Yes | Yes | Yes | Yes | Yes | Yes | Yes |
| 8 | NA | NA | NA | NA | NA | NA | NA | NA | NA | NA | NA | NA | NA | NA | Yes | No | NA | NA |
| 9 | No | No | No | No | No | No | No | No | No | Yes | NTD | No | No | No | Yes | No | No | No |
| 10 | Yes | Yes | Yes | Yes | Yes | Yes | Yes | Yes | Yes | Yes | Yes | Yes | Yes | Yes | Yes | Yes | Yes | Yes |
| 11 | No | Yes | Yes | Yes | No | No | No | Yes | Yes | Yes | No | No | No | Yes | Yes | Yes | Yes | Yes |
| 12 | No | No | No | Yes | No | No | No | Yes | Yes | Yes | No | Yes | No | Yes | Yes | Yes | Yes | No |
| 13 | Yes | Yes | Yes | Yes | Yes | Yes | Yes | Yes | Yes | Yes | Yes | Yes | Yes | Yes | Yes | Yes | Yes | Yes |
| 14 | NA | NA | NA | NA | NA | NA | Yes | NA | NA | NA | NA | NA | NA | NA | No | NA | NA | NA |
| 15 | No | No | No | No | No | No | Yes | No | No | No | No | Yes | Yes | No | No | No | No | No |
| 16 | NA | No | NA | NA | NA | Yes | NA | NA | NA | NA | No | No | No | NA | No | No | No | No |
| 17 | No | Yes | Yes | Yes | Yes | Yes | Yes | Yes | Yes | Yes | Yes | Yes | Yes | No | No | Yes | Yes | Yes |
| 18 | Yes | Yes | Yes | Yes | Yes | Yes | Yes | Yes | Yes | Yes | Yes | Yes | Yes | Yes | Yes | Yes | Yes | Yes |
| 19 | NA | NA | NA | NA | NA | NA | NA | NA | NA | NA | NA | NA | NA | NA | Yes | NA | NA | NA |
| 20 | Yes | Yes | Yes | Yes | Yes | Yes | Yes | Yes | Yes | Yes | Yes | Yes | Yes | Yes | Yes | Yes | Yes | Yes |
| 21 | Yes | Yes | Yes | Yes | Yes | Yes | Yes | Yes | Yes | Yes | Yes | Yes | Yes | No | Yes | Yes | Yes | Yes |
| 22 | Yes | Yes | Yes | Yes | Yes | Yes | Yes | Yes | Yes | Yes | Yes | Yes | Yes | No | Yes | Yes | Yes | Yes |
| 23 | NA | NA | NA | NA | NA | NA | NA | NA | NA | NA | NA | NA | NA | NA | No | NA | NA | NA |
| 24 | No | No | No | No | No | No | No | No | Yes | No | No | Yes | Yes | Yes | Yes | No | No | No |
| 25 | No | No | No | No | No | Yes | Yes | No | Yes | Yes | Yes | Yes | No | No | No | No | No | No |
| 26 | UTD | Yes | UTD | UTD | UTD | UTD | UTD | UTD | UTD | Yes | UTD | UTD | UTD | UTD | Yes | UTD | UTD | UTD |
| 27 | No | No | No | No | NO | No | No | No | No | No | No | No | No | No | No | No | No | No |

Legend - NA: non-applicable, UTD: unable to determine
