# Supplementary material for: Prognostic relevance of exercise testing in hypertrophic cardiomyopathy. A systematic review
Source: Int J Cardiol. 2021 Sep 15;339:83–92. doi: 10.1016/j.ijcard.2021.06.051 (PMC8425182; doi:10.1016/j.ijcard.2021.06.051)
Supplement: Supplementary material 1 — Supplementary methods. [file mmc5.docx]

**SUPPLEMENTARY METHODS**

**Resting and exercise echocardiography**

Parameters considered of relevance for analysis were left ventricular (LV) maximal wall thickness (mm), LV ejection fraction (LVEF) (%), LVOT gradient (mmHg), systolic anterior motion (SAM) of the mitral valve, mitral regurgitation (MR), left atrial (LA) diameter (mm) and LA indexed volume (mm/m²), regional wall motion abnormalities (WMA).

Some of the parameters measured in resting echocardiography, such as LVOT gradient, WMA and MR were measured during exercise, including at peak, and after the test.

**Cardiopulmonary exercise testing**

Parameters of interest were VO2 (mL/Kg/min); VE/VCO2 slope; peak respiratory exchange ratio (RER), defined as the ratio between VCO2 and VO2 during peak exercise; and anaerobic threshold, calculated using the V-slope method.
